# GLTP Mediated Non-Vesicular GM1 Transport between Native Membranes

**DOI:** 10.1371/journal.pone.0059871

**Published:** 2013-03-28

**Authors:** Ines Lauria, Jan van Üüm, Esmina Mjumjunov-Crncevic, David Walrafen, Luis Spitta, Christoph Thiele, Thorsten Lang

**Affiliations:** 1 Department of Membrane Biochemistry, LIMES (Life and Medical Sciences) Institute, University of Bonn, Bonn, Germany; 2 Department of Biochemistry and Cell Biology of Lipids, LIMES Institute, University of Bonn, Bonn, Germany; Institut Curie, France

## Abstract

Lipid transfer proteins (LTPs) are emerging as key players in lipid homeostasis by mediating non-vesicular transport steps between two membrane surfaces. Little is known about the driving force that governs the direction of transport in cells. Using the soluble LTP glycolipid transfer protein (GLTP), we examined GM1 (monosialotetrahexosyl-ganglioside) transfer to native membrane surfaces. With artificial GM1 donor liposomes, GLTP can be used to increase glycolipid levels over natural levels in either side of the membrane leaflet, i.e., external or cytosolic. In a system with native donor- and acceptor-membranes, we find that GLTP balances highly variable GM1 concentrations in a population of membranes from one cell type, and in addition, transfers lipids between membranes from different cell types. Glycolipid transport is highly efficient, independent of cofactors, solely driven by the chemical potential of GM1 and not discriminating between the extra- and intracellular membrane leaflet. We conclude that GLTP mediated non-vesicular lipid trafficking between native membranes is driven by simple thermodynamic principles and that for intracellular transport less than 1 µM GLTP would be required in the cytosol. Furthermore, the data demonstrates the suitability of GLTP as a tool for artificially increasing glycolipid levels in cellular membranes.

## Introduction

Plasmalemmal and intracellular membranes vary in their unique protein and lipid compositions [1], [2]. Classically, it is assumed that metabolism and vesicle trafficking generate the observed membrane diversity and control lipid homeostasis. However, lipid transfer proteins (LTPs) have emerged as novel key players mediating non-vesicular lipid transport steps [3]–[5].

LTPs can be genetically classified or functionally sub-divided into three major groups: phospholipid-, sterol- and sphingolipid-transfer proteins [4]. All LTPs contain a lipid binding domain and often additional motifs for subcellular targeting. In a few cases, the lipid binding domain is also able to recognize several related lipids. Though LTPs have an intrinsic lipid transfer activity, some of them might play a role as lipid sensor [6], [7].

It seems clear that LTPs mediate the transport of lipids between the surfaces of different intracellular membranes, yet it is a matter of debate how directionality of transport is achieved. For instance, the ceramide transporter Cert/STARD11 and the glycolipid transfer protein FAPP2 act at specific membrane contact sites. According to the “short-distance shuttle” and “neck-swinging” models, Cert binds to Golgi and ER membranes via its pleckstrin homology (PH) domain and two phenylalanines in an acidic tract (FFAT)-motif, respectively [8]. Assisted by its targeting domains, Cert is capable of transporting newly synthesized ceramide efficiently from the ER to the Golgi. Similarly, membrane associated FAPP2 functions at ER-Golgi contact sites. Here, it binds glucosylceramide, synthesized at the cytosolic surface of the Golgi, and incorporates it into the ER [9]. Alternatively, FAPP2 might mediate transport between *cis*- and *trans*-Golgi membranes [10].

Not all LTPs possess a targeting domain, indicating that association with specific membranes is not a prerequisite for non-vesicular lipid trafficking. For instance, overexpression of the soluble cholesterol binding protein STARD4 provides indirect evidence for elevated cholesterol transport to the ER and mitochondria [11]–[14]. However, it remains unclear whether LTPs exhibit an intrinsic preference for insertion into specific acceptor membranes or whether posttranslational modifications, interactions with other proteins or the conformation of the LTP itself govern the recognition of the target membrane [15]. Alternatively, transfer could be solely driven by the chemical potential, in the case of STARD4 resulting in cholesterol transport from e.g. the plasma membrane to low cholesterol level compartments such as the ER or mitochondria.

The glycolipid transfer protein (GLTP) is another example for a soluble, cytosolic LTP [7], [16], [17], though identification of a FFAT-like motif could point to an association with the ER [18]. Its transfer activity was discovered in 1980, reporting that a partially purified protein transports radioactive glucosylceramide from liposomes to red blood cells [19]. It has been suggested that GLTP functions in intracellular glucosylceramide trafficking [16]. In fact, GLTP knock-down experiments show a 50% reduction of glucosylceramide transport to the plasma membrane while GLTP-overexpression, in the absence of vesicular trafficking, results in a more than two-fold increase of transport [9]. However, glucosylceramide still reaches the plasma membrane in the absence of GLTP and vesicular transport, consistent with transport mediated by FAPP2 [9]. In an alternative model, based on the observation that release of glycolipids into phosphatidylcholine acceptor vesicles is inefficient [20], GLTP assumes the role of a lipid sensor. Moreover, overexpression of GLTP influences the levels of glucosylceramide and sphingomyelin [7] and human *GLTP* gene expression is regulated by ceramide levels [21] linking GLTP to lipid biosynthesis and homeostasis. In conclusion, the exact biological role of GLTP still remains elusive.

Several studies have characterized GLTP accelerated transfer of glycolipids between artificial bilayers. Under these conditions, GLTP recognizes and transfers various glycolipids composed of a ceramide or glycerolipid backbone with a β-linked sugar residue, including glucosylceramide, galactosylceramide, lactosylceramide and gangliosides like GM1, asialo-GM1, GD1a, GD1b or GT1b [22]–[27]. The transfer cycle in artificial membrane systems is limited by the kinetics of the depletion reaction [28] and depends on membrane curvature [29] as well as lipid composition [30], [31]. In particular, extraction of glycolipids becomes inefficient when the donor membrane contains sphingolipids, presumably due to tight glycolipid-sphingolipid clustering into microdomains [30].

Here, we have systematically studied GLTP transport from/to native membranes. Compared to artificial membrane systems, native membranes are densely crowded with membrane proteins and provide natural lipid mixtures and concentrations. Therefore, they allow addressing the following open questions: First, does membrane crowding still enable efficient transfer cycles? Second, what is the lipid loading capacity of a native membrane? Saturation could be reached particularly quickly for more bulky glycolipids. Third, what GLTP concentration is necessary for efficient transfer? Fourth, would the concentration gradient between native membranes be a sufficient driving force for lipid transport? To address these questions we have selected to study the transfer of the glycolipid GM1 since it has a large head-group and can be quantified reliably over a large concentration range by cholera toxin (CTX) binding [27].

## Results

**Figure 1:**
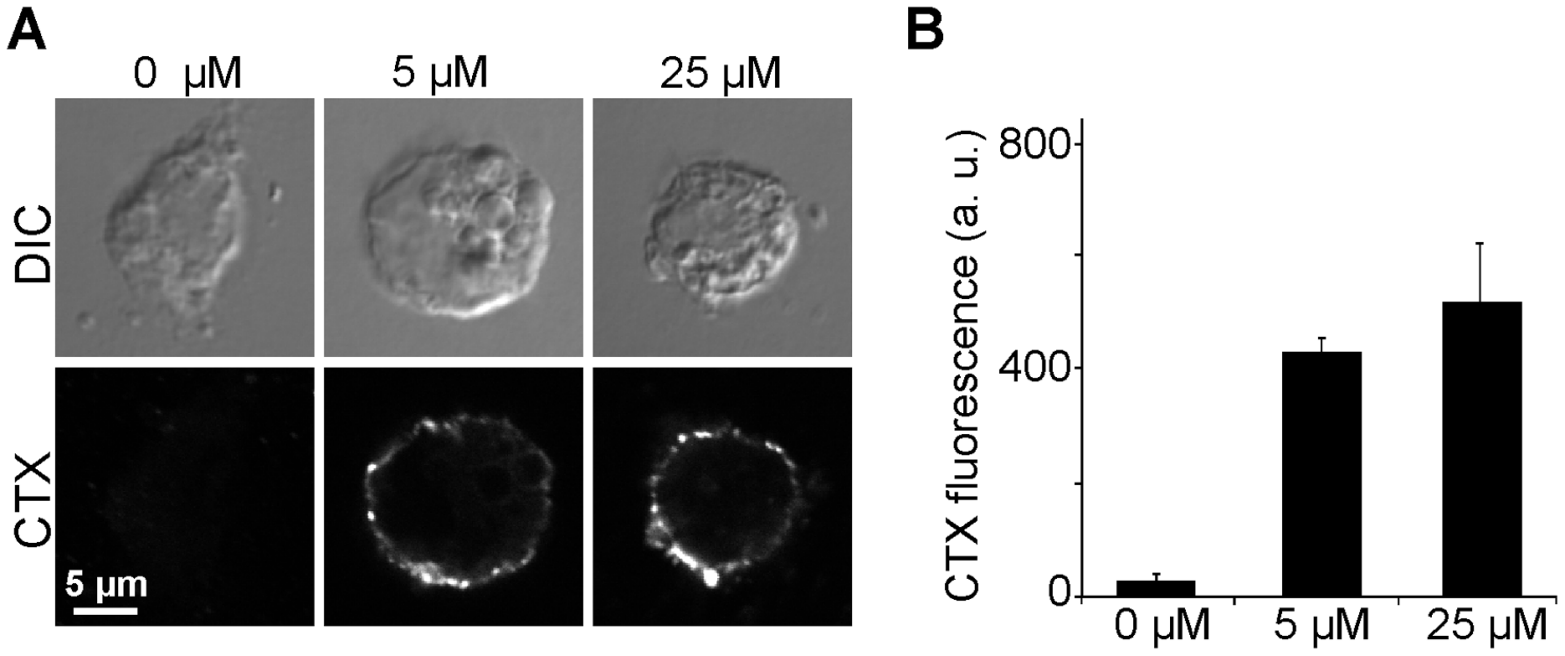
GLTP readily inserts GM1 into the extracellular leaflet of HepG2 cells. (A) HepG2 cells were incubated at 37°C for 10 min in a 20 min pre-incubated Ringer solution containing 0.2 mg/ml GM1-DOPC (5∶95 mol%)-liposomes with 0, 5, or 25 µM GLTP. Then cells were washed, stained in the cold with fluorescently labeled CTX, fixed and imaged. Pictures were recorded with a confocal laser scanning microscope in the differential interference contrast (DIC) and the fluorescence channel. Images in the lower panels are shown at the same scaling leading to the impression that in control cells staining is missing. However, also in control cells staining was present in the periphery (see Fig. S2A). (B) CTX staining intensity at the cell periphery was quantified by linescan analysis (three pixel width). Values are given as means ± SEM (n  = 3 - 4 independent experiments; 19 - 35 cells were analyzed for each experiment).

First, we tested the efficiency of GLTP-mediated transfer of GM1 from liposomes into native plasma membranes. To this end, GLTP was pre-incubated with liposomes containing 5 mole percent GM1 for 20 min at 37°C, followed by addition of the mixture to HepG2 cells. After 10 min incubation we probed the plasmalemmal GM1 content with fluorescent CTX, which was added to live cells in the cold to avoid endocytosis. Then, cells were washed, fixed and the distribution of CTX fluorescence was analyzed in the equatorial plane of the cell. As expected, a peripheral staining pattern was observed both in control and GLTP treated cells (Fig. 1A). At 5 µM, GLTP produced a 16-fold brighter staining (Fig. 1B). Further elevation of GLTP to 25 µM increased CTX staining only modestly, indicating that the loading capacity of the outer membrane leaflet started to saturate. Hence, native membranes have a large capacity for taking up additional glycolipids albeit it cannot be excluded that due to the broad specificity of GLTP for glycolipids the increase reflects in part also exchange of glycolipids by GM1.

**Figure 2:**
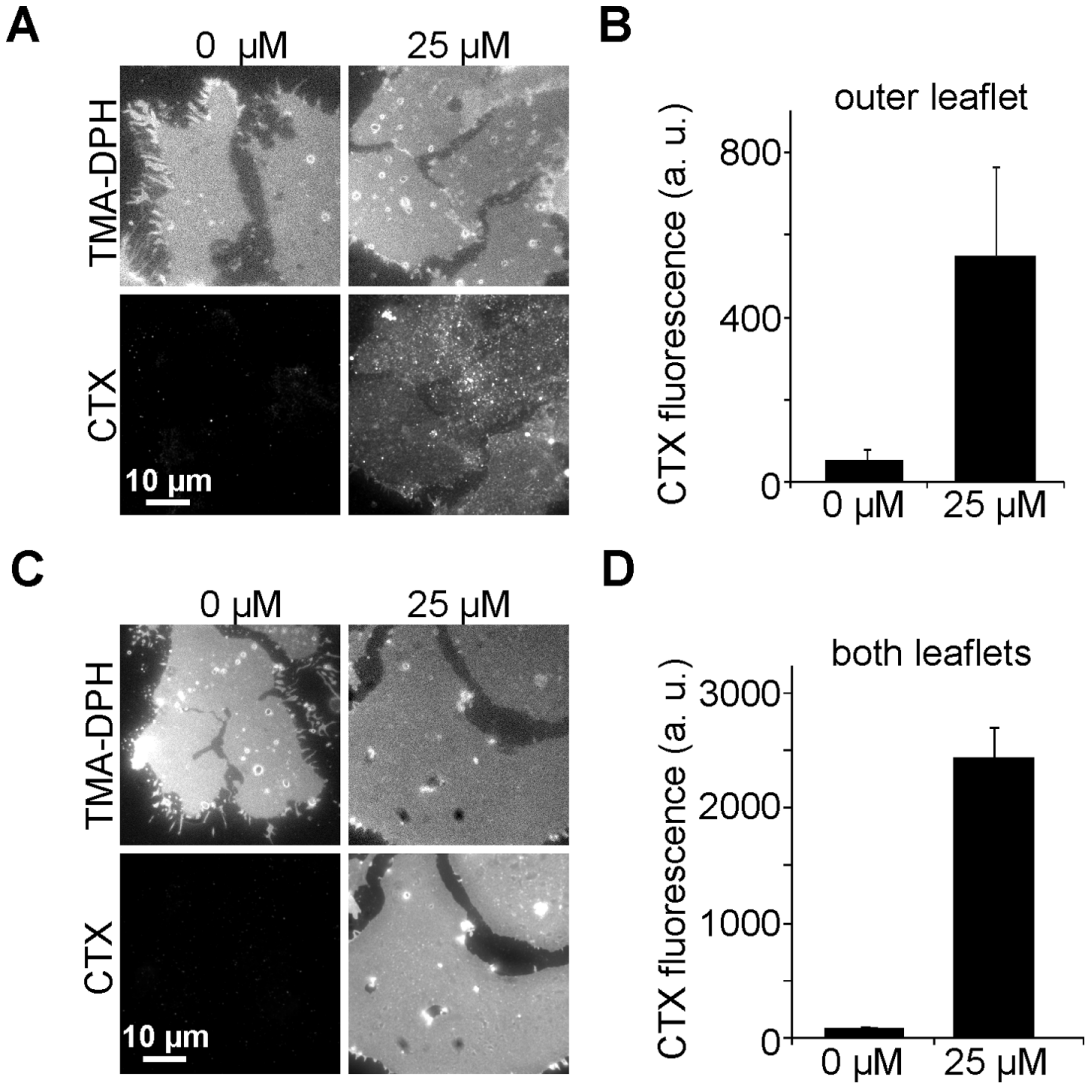
Insertion into the intra- and the extracellular plasmalemmal leaflet. (A) For GM1 loading HepG2 cells were treated as in Fig. 1. Then membrane sheets were generated and CTX staining was performed at RT. Samples were fixed and basal plasma membranes were imaged in the TMA-DPH (general membrane staining) and CTX channel. (B) Quantitation of the mean CTX fluorescence intensities reflecting the GM1 increase in the extracellular leaflet. Values are given as means ± SEM (n  = 3 independent experiments; 18 - 50 membrane sheets analyzed for each experiment). (C and D) For incorporation of GM1 into both membrane leaflets, membrane sheets were generated prior to the incubation with GLTP/liposomes, allowing biochemical access to the inner plasmalemmal leaflet. After GM1 insertion, GM1 staining, imaging and quantification of fluorescence was performed as in A and B. In this experiment fluorescence arises from both leaflets; fluorescence from the inner plasmalemmal leaflet has a more uniform appearance (Fig. 3). Values are given as means ± SEM (n  = 4 - 5 independent experiments; 36 - 53 membrane sheets analyzed for each experiment).

To reveal any insertion preference for differently composed membrane surfaces we turned to plasma membrane sheets. Plasma membrane sheets are generated from substrate-adhered cells subjected to a brief ultrasound-pulse which removes the upper parts of the cells while leaving the intact basal plasma membranes adhered to the substrate [32], [33]. The preparation is essentially two-dimensional enabling imaging with a conventional fluorescence microscope without the need for removal of out of focus light producing high signal-to-noise recordings of the spatial distribution of the CTX staining. More importantly, on plasma membrane sheets both different leaflets are biochemically accessible for GM1 insertion. The topology of the two leaflets differs in several aspects. Apart from the specific protein domains, the inner plasmalemmal leaflet is more negatively charged and crowded with cytoskeletal elements, whereas the outer leaflet has a dense glycocalyx. To obtain a reference for insertion into the extracellular leaflet only, we treated whole cells as in Fig. 1. but quantified fluorescence on plasma membrane sheets. With 25 µM GLTP we found an 11-fold increase of staining (Fig. 2A and B). Compared to Fig. 1, the lower relative increase might indicate that insertion into the basal membrane is limited by diffusion, or that the absolute GM1 level in the basal plasma membrane, to which the increase is related, is higher. Then we loaded both the extracellular and intracellular plasmalemmal leaflets by incubating membrane sheets with GLTP/liposomes. As illustrated in Fig. 2C and D, CTX-staining was elevated 28-fold, which reflects the sum of the increases in each of the opposed leaflets, indicating that the increase in the inner leaflet is 17-fold. Under this experimental configuration, to exclude that GM1 increase results from other processes than GLTP transfer activity, we also tested the GLTP-W96A mutant which is severely less able to recognize glycolipids [25] and the mutant W142A with a strongly diminished ability to bind to membranes [34]. As expected, the mutations strongly inhibited transfer activity (Fig. S1).

**Figure 3:**
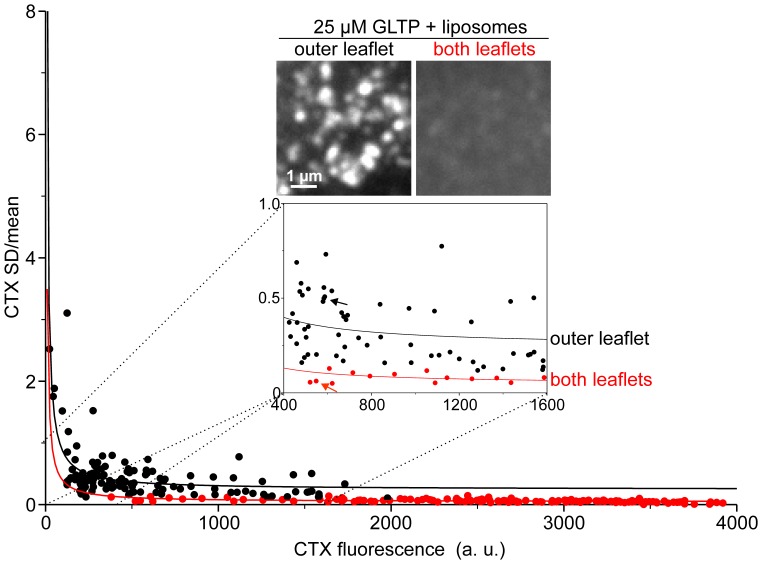
GM1 in the inner leaflet is more uniformly distributed. The degree of clustering of the CTX staining was analyzed on membrane sheets from experiments shown in Fig. 2 by calculating the relative SD (SD/mean). For each membrane sheet, SD/mean was plotted against the mean fluorescence and a function (*y* = *y_0_*+ *a*/*x*) was fitted (with values for “outer leaflet” *y_0_* = 0.2437; *a*  = 62.0138 and for “both leaflets” *y_0_* = 0.0454; *a*  = 34.49). The higher the value of the relative mean, at a comparable GM1 concentration, the more clustered the signal [39]. Loading of the outer leaflet (black dots) results in overall higher values than loading of both leaflets (red dots). Arrows point to the values obtained for the membranes shown for illustration. For each condition, membrane sheets from all independent experiments were pooled (128 and 140 membrane sheets for loading of the extracellular and both leaflets, respectively).

Apparently, as suggested by Fig. 2, insertion into the inner leaflet is slightly more efficient, possibly due to better accessibility. The finding could be also explained by the observed different lateral organization of the newly inserted GM1 molecules. In line with the idea of tight GM1-sphingolipid clustering into microdomains, GM1 in the outer leaflet has a strongly clustered appearance, whereas fluorescence in the inner leaflet was more evenly distributed (Fig. 3), which could be associated with a better accessibility for CTX staining. In any case, the data clarifies that GLTP very efficiently inserts GM1 into native membranes and has no strong preference for one leaflet. Hence, it appears that GLTP membrane interactions are promiscuous and do not involve cofactors. Most likely, as shown on artificial membranes, GLTP binding requires, along with nearby nonpolar residues, a specific Tryptophan residue acting as a shallow-penetration anchor [35] and occurs even in the absence of glycolipids [31]. Furthermore, the data show that the cell membrane of HepG2 cells has a large capacity for taking up additional glycolipid molecules, more than 1000% per leaflet, suggesting that natural glycolipid contents are not a result of glycolipid saturation.

**Figure 4:**
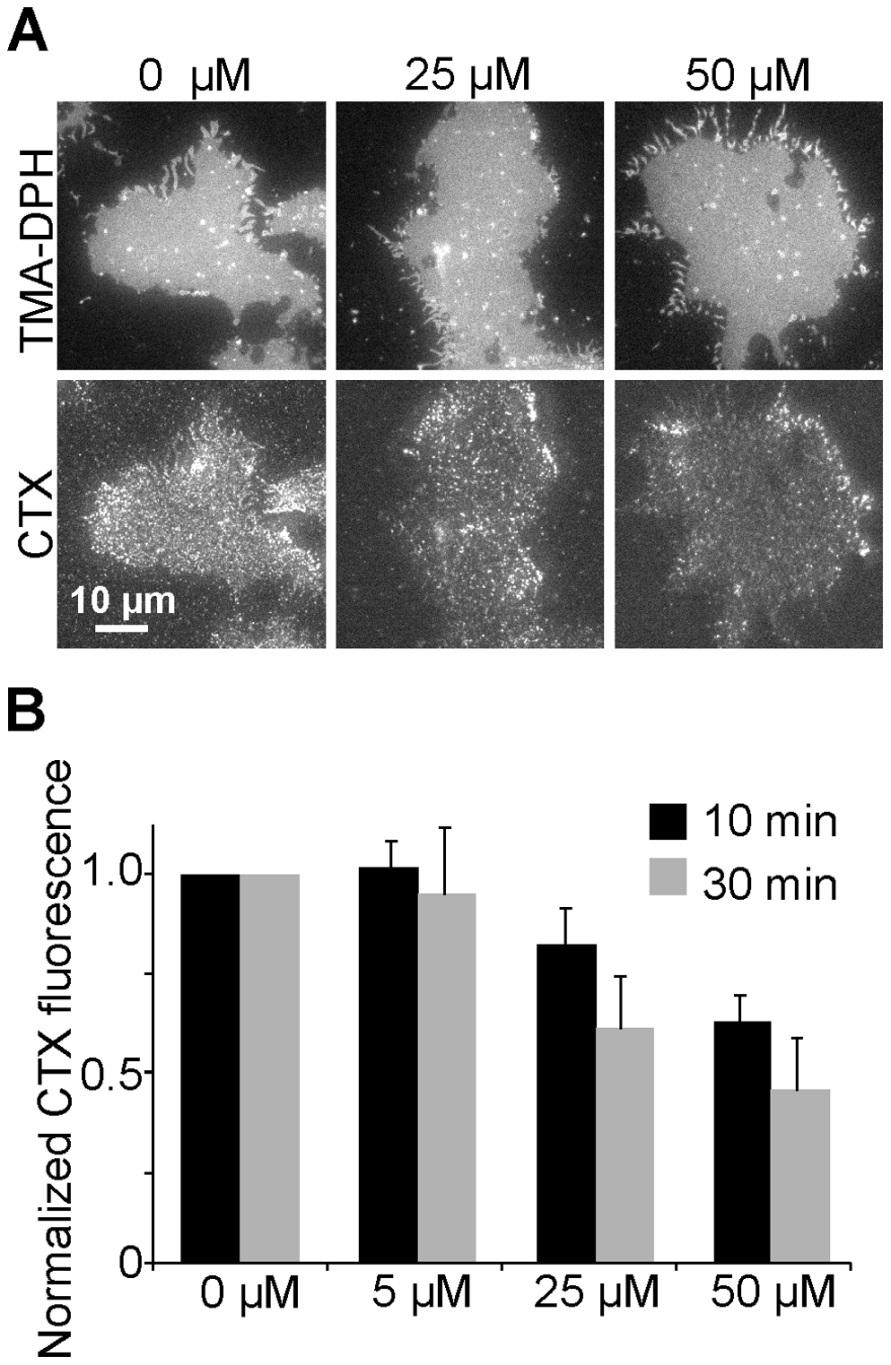
GM1 extraction from plasma membrane sheets. (A) Membrane sheets were generated from HepG2 cells, incubated with GLTP at variable concentrations for 10 min (not shown) or 30 min at 37°C, washed, stained at RT with fluorescent CTX, fixed and imaged in the TMA-DPH and the CTX channel. (B) Quantitative analysis of mean CTX staining intensities, normalized to control values. GLTP decreases the GM1 staining in a time and concentration dependent manner though even under the harshest condition GM1 was reduced to only 45%. Values are given as means ± SEM (n  = 4 - 5 independent experiments; 8 - 81 membrane sheets analyzed for each experiment).

**Figure 5:**
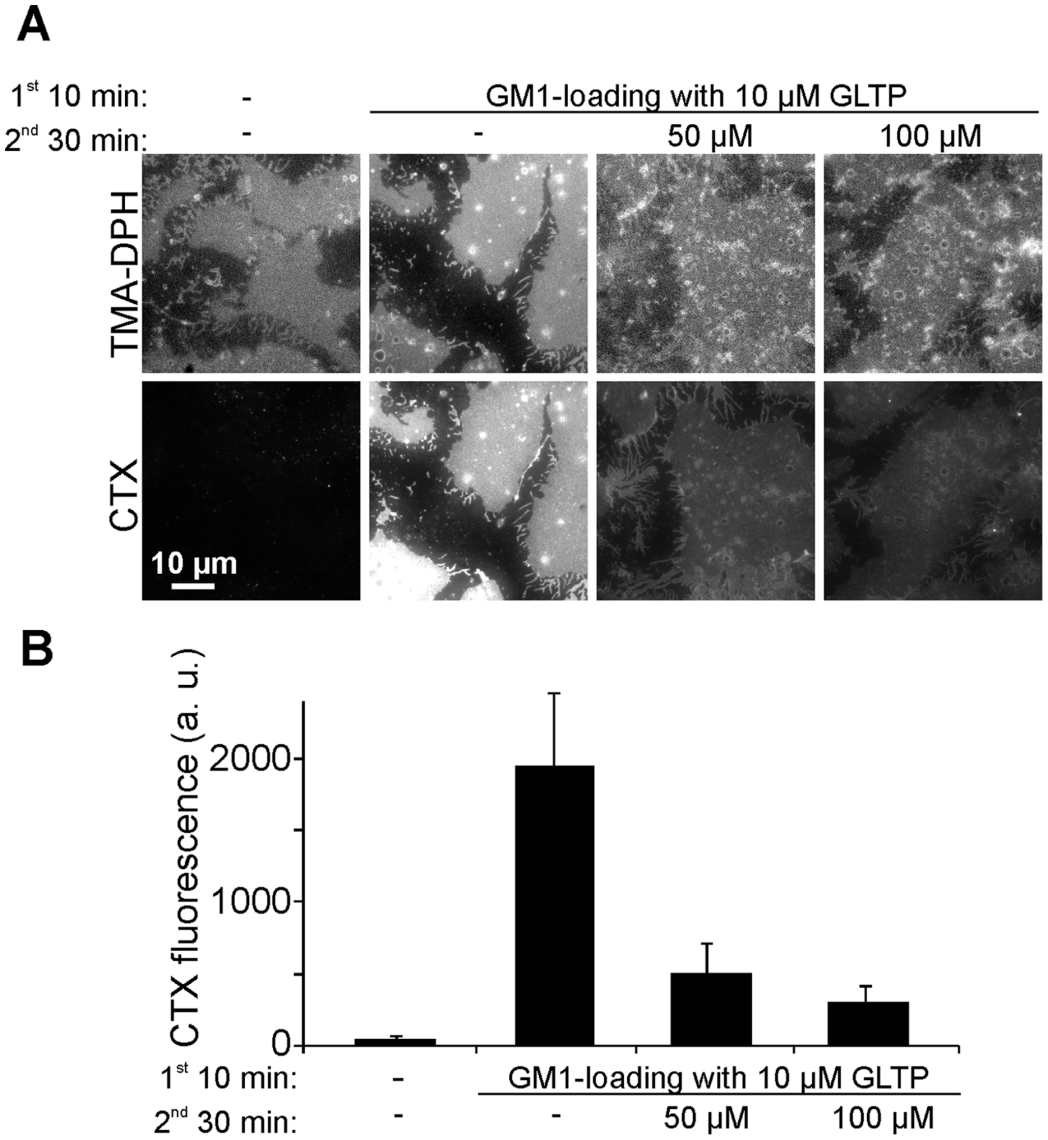
Extraction of inserted GM1. (A) HepG2 cell membrane sheets were loaded at 37°C for 10 min with 10 µM GLTP/liposomes (in the control GLTP was omitted) followed by a second incubation at 37°C for 30 min with 0, 50 or 100 µM GLTP. (B) Quantification of fluorescence in the CTX channel. Values are given as means ± SEM (n  = 3 independent experiments; 36 - 68 cells were analyzed for each experiment).

**Figure 6:**
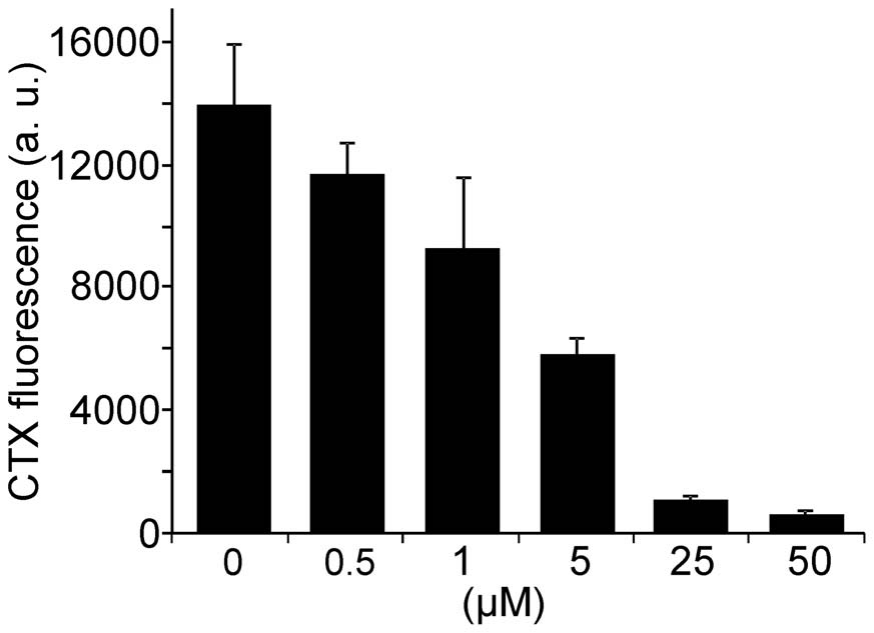
Extraction of GM1 from Jurkat cell membrane sheets. Jurkat cell membrane sheets were incubated at 37°C for 30 min with variable concentrations of GLTP and fluorescence in the CTX channel was quantified. The bar chart includes also data shown in Fig. 8. Values are given as means ± SEM (n  = 3 - 5 independent experiments; 142 - 316 cells were analyzed for each experiment).

Previously, GLTP has been applied for depletion of glycolipids from native cell membranes. Various cell types were treated for 45 min at 37°C with 1.5 mg/ml GLTP (corresponding to approximately 60 µM) which depleted 40% glucosylceramide and 45% GM3 [9]. When we applied GLTP to HepG2 cells, even 30 min treatments with 100 µM GLTP hardly reduced GM1 levels (Fig. S2). Here, we may underestimate the extraction efficiency since many cells, probably those most affected by GLTP, were lost from the coverslip and therefore escaped the microscopic analysis. To verify the GLTP effects in the absence of cell detachment, we first produced membrane sheets and then incubated with GLTP. As shown in Fig. 4, CTX staining was decreased in an incubation time and GLTP concentration dependent manner. The maximal reduction to 45% was observed with 50 µM GLTP for 30 min. Hence, the extraction efficiency of GM1 is similar to the values reported for glucosylceramide and GM3. However, depletion should not only depend on the GLTP concentration but on the concentration of glycolipids available in the membrane and whether the reaction has reached chemical equilibrium. To explore this possibility in more detail, we artificially increased GM1 in HepG2 cells and then added GLTP for 30 min (Fig. 5). Under these conditions, HepG2 membrane sheets start with a 46-fold higher GM1 concentration what leads to a stronger relative depletion (Fig. 5). To validate this for naturally occurring high GM1 levels we used membrane sheets from Jurkat cells with a 74-fold higher endogenous GM1 concentration compared to HepG2 cells. As depicted in Fig. 6, depletion was very efficient, resulting in a reduction to less than 5%. We conclude that the fraction of GM1 kept in solution (bound to GLTP) increases with the level of GM1 available in the membrane.

**Figure 7:**
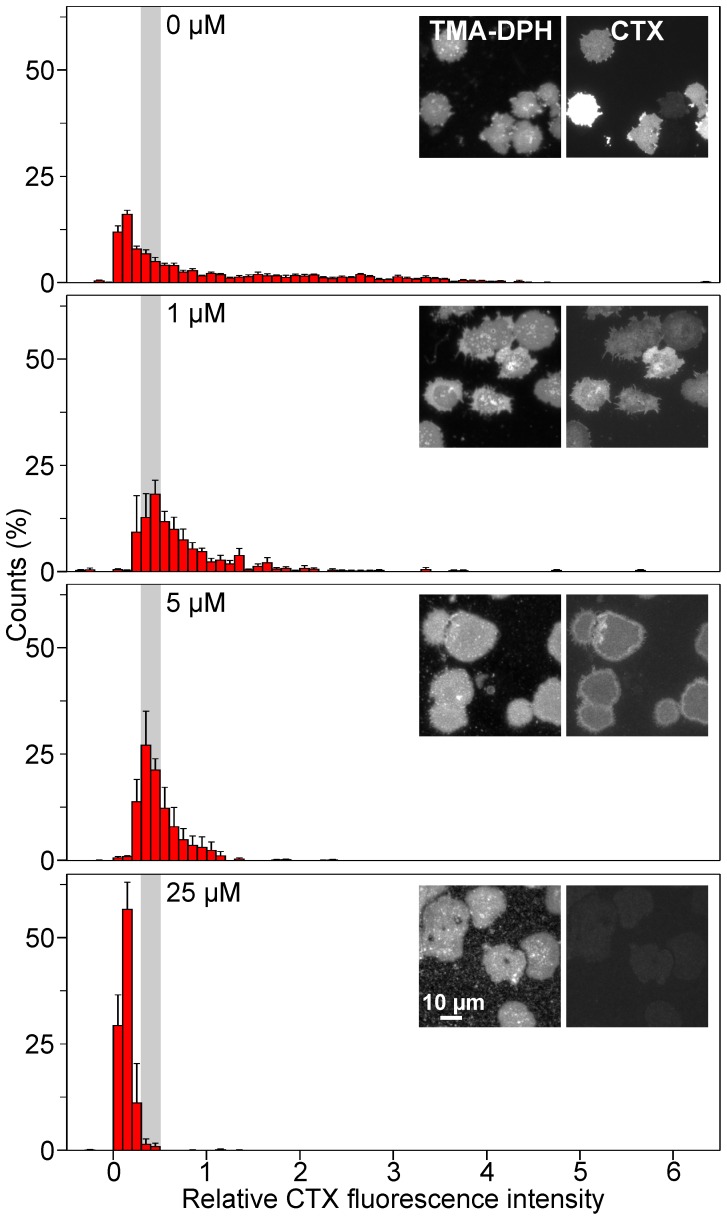
Micromolar concentrations of GLTP balance GM1 concentrations between Jurkat cell membrane sheets. Jurkat cell membrane sheets were incubated at 37°C for 30 min with 0, 1, 5 or 25 µM GLTP, followed by staining with CTX and imaging. For each condition images from the TMA-DPH and the CTX channel are shown. CTX fluorescence intensity values from individual membrane sheets were normalized to the average intensity of the control and values were plotted as a histogram. At 1 and 5 µM a shift of the distribution peak to higher fluorescence values is observed. Fig. 6 shows the average intensity remaining after GLTP treatment. Values are given as means ± SEM (n  = 3 - 5 independent experiments; 142 - 316 membrane sheets analyzed for each experiment).

In our experimental system native membranes are at the same time putative donors and acceptors. We hypothesized that in a population of individual membranes with naturally variable GM1 levels, GM1 rich membranes act more likely as donors and those with sparse GM1 as acceptors. In equilibrium, GLTP should have balanced the concentration differences, assuming all factors relevant for the chemical potential of GM1 being similar in all membranes. To test this, we incubated Jurkat cell membrane sheets with variable GLTP concentrations and plotted individual membrane sheet intensities in a histogram (Fig. 7). Control membrane sheets vary largely, with individual membranes being more than three-fold brighter than the average intensity. At 1 µM GLTP, membrane sheets with high GM1 content diminish while the population with half of the average control intensity grows. The narrowing of the intensity distribution is clearer for 5 µM at which the very dark and bright membranes disappear. The effect cannot be produced solely by depletion from bright membranes (see Fig. 6 for the average depletion effects), as within the population of darker membranes the peak shifted from 0.2 to 0.4. Thus, membranes with sparse GM1 have received GM1 from GM1 rich membranes, or in other words, GLTP has balanced the different concentrations between individual membrane sheets.

**Figure 8:**
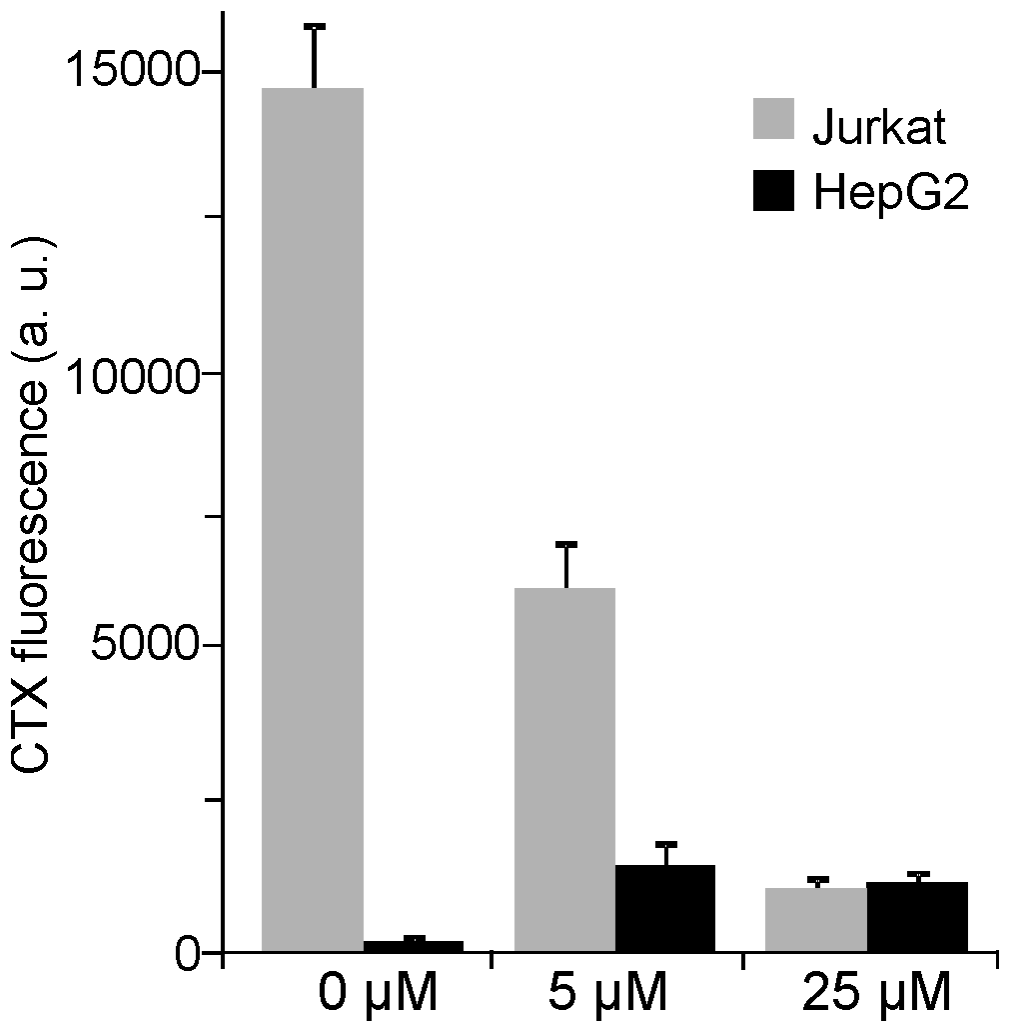
Transfer of Jurkat cell GM1 to membrane sheets from HepG2 cells. GLTP solutions from Jurkat cell membrane treatments (Fig. 7; 30 min at 37°C) were collected and added to HepG2 membrane sheets. After 30 min at 37°C GM1 was visualized by CTX staining and imaged. The bar chart represents quantified CTX fluorescence of Jurkat cell membranes (from the experiment shown in Fig. 7) and HepG2 cell membranes. Jurkat cell membranes and HepG2 membranes were recorded with 100 ms and 1 s, respectively. To allow a direct comparison of the GM1 levels, the presented HepG2 values were divided by a factor of 10 assuming that the recorded intensity is linear to the exposure time. In controls, Jurkat cells had a 74-fold higher GM1 concentration than HepG2 cells. Values are given as means ± SEM (n  = 3 independent experiments; 113 - 316 membrane sheets analyzed for each experiment).

At 25 µM GLTP, GM1 in the membranes has diminished to 8% (Fig. 6), indicating that the large majority of the Jurkat cell GM1 molecules is kept in solution, bound to GLTP. The factors which determine the GM1 chemical potential should be similar in Jurkat and HepG2 cells. In this case, the solution resulting from Jurkat cell membrane extraction is suitable for loading membranes from HepG2 cells since the GM1 content of Jurkat cells after treatment still exceeds the physiological GM1 levels in HepG2 cells several-fold. Indeed, solutions from Jurkat cell membrane extractions inserted their solubilized GM1 into HepG2 cell membranes, resulting in GM1 levels similar to the one in depleted Jurkat cell membranes (Fig. 8).

## Discussion

### GLTP Mediated Transport is Driven only by Concentration Gradients

Our study shows that GLTP readily inserts GM1 from liposomes into both leaflets of a native plasma membrane, demonstrating that membrane crowding does not preclude efficient transfer cycles.

When HepG2 membrane sheets with low GM1 levels were incubated with GLTP/liposomes, as little as 5 µM GLTP was sufficient to increase the levels manifold above physiological values within 10 min. In contrast, 30 min with 50 µM GLTP resulted in a reduction to only 45%. At first glance, in line with previous reports using artificial membrane systems [28] it appears that insertion is more efficient than depletion. However, as Fig. 8 shows, the net transfer and accordingly the specific depletion and insertion rates depend strongly on the GM1 concentrations in the acceptor and donor membranes and the concentration of GLTP. Therefore we conclude that GLTP mediated transport is driven only by concentration gradients. Moreover, as in our system the cytosol has been removed and GM1 is also readily inserted into the outer membrane leaflet, we propose that transport is independent of any intracellular cofactors.

### The Physiological Role of GLTP

The data also shows that within 30 min 1 µM GLTP is sufficient to diminish physiological GM1 concentration gradients. Considering that in our assay system for the completion of one transfer cycle GLTP diffuses over large distances between membrane sheets, much less GLTP would be required to degrade concentration gradients between membrane surfaces in the cytosol of a live cell. As the binding affinities of GLTP for GM1 and glucosylceramide are similar [26], the data indicates that *in vivo* GLTP could transfer efficiently glycolipids like glucosylceramide between the cytosolic surfaces of intracellular membranes solely driven by concentration gradients. However, our data does not exclude a role of GLTP in glycolipid sensing.

### Using GLTP as Tool for Glycolipid Level Manipulation

The capability to readily insert glycolipids into native membranes opens the possibility to apply GLTP as a biochemical tool. Glycosphingolipids are crucial components of cell membranes with a multitude of functions in life and disease [36]–[38]. To study the role of glycolipids or to administer glycolipids, means for increasing glycolipid levels in the outer cell membrane would be highly desirable. Genetic down- or up-regulation of lipid synthesis or turnover can cause secondary effects due to accumulation or depletion of precursors or subsequent components in the biosynthetic cascade rendering a more direct method for glycolipid insertion very attractive. Here, we have established such an application of GLTP to native membranes. As the glycolipid levels can be manifold increased above control levels, we suggest that GLTP is a powerful tool for increasing acutely and specifically the concentration of GM1 or maybe other glycolipids in native membranes.

## Materials and Methods

### Cell Culture

HepG2 cells were maintained essentially as described previously [39]. One day before the GLTP treatment 1.5 - 3 x 10^5^ cells per 6-well plate were seeded onto 25 mm^2^ poly-L-lysine coated cover slips. Jurkat cells E6.1 (Cat#88042803, Sigma) were grown in suspension in 175 cm^2^ flasks in RPMI1640 supplemented with penicillin (100 U/ml)/streptomycin (0.1 mg/ml) (PAA) and 10% FBS-Superior (standardized fetal bovine serum, Biochrom, Berlin, Germany). 2.5 x 10^6^ cells were adhered to one coverslip in 30 min at 37°C in Ringer solution (130 mM NaCl, 4 mM KCl, 1 mM CaCl_2_, 1 mM MgCl_2_, 48 mM D(+)glucose, 10 mM HEPES-NaOH, pH 7.4).

### Cloning and Expression of GLTP

For N-terminal hexa-histidine tagging of human GLTP the open reading frame (GenBank: AY372532.1) was amplified by RT-PCR from HepG2 total RNA (Cat# 210210, OneStep RT-PCR Kit, QIAGEN, Hilden, Germany) using the oligonucleotides 5′-*Nde*I-GCGCTGCTGGCCGAACACTTGCT-3′ and 5′-*Bam*HI-CTACACCTTGTAGTTGAGCTC-3′, subcloned into pGEM-T Easy (Cat#A1360, Promega) and ligated into the expression vector pET-15b (Cat# 69661, Merck, Darmstadt, Germany). The GLTP mutants W96A and W142A were generated by fusion PCR with overlapping primers carrying the point mutations (for W96A the primers 5′-GCGCTGATGgcgCTGAAAAGA-3′ and 5′-TCTTTTCAGcgcCATCAGCGC-3′ and for W142A the primers 5′-TACCATGGCgcgATCGTGCAG-3′ and 5′-CTGCACGATcgcGCCATGGTA-3 were used, respectively). After subcloning into the pGEM-T Easy vector the constructs were ligated into the pET-15b expression vector via the *NdeI* and *BamHI* restriction sites.

After transformation into BL21(DE3)pLysS (Cat# L1191, Promega) soluble GLTP and GLTP mutants were expressed after induction with 10 mM IPTG for 6 h at 24°C. Bacterial pellets were sonicated in NiNTA-buffer (50 mM NaH_2_PO_4_, 150 mM NaCl, 1 x complete EDTA free (Roche, Mannheim, Germany), 1 mM PMSF, pH 7) containing 1% [v/v] Triton X-100, 10% [v/v] glycerol, 10 mM β-mercaptoethanol and 100 µg/ml lysozyme (Carl Roth, Karlsruhe, Germany). Cleared lysates were further bound to equilibrated Protino NiNTA Agarose (Macherey-Nagel, Dueren, Germany) for 2 h. After several washing steps with NiNTA-buffer containing increasing concentrations of up to 70 mM imidazole, GLTP was eluted with 300 mM imidazole. The buffer was exchanged to PBS (137 mM NaCl, 2.7 mM KCl, 10 mM Na_2_HPO_4_, 1.76 mM KH_2_PO_4_) containing 10% [v/v] glycerol using Amicon Ultra-15 (membrane 10 kDa, Millipore). Aliqoted samples were frozen in liquid nitrogen and stored at −80°C.

### GLTP Treatments of Cells and Membrane Sheets

GLTP was diluted – when incubated with whole cells – in Ringer solution or – when incubated with membrane sheets – in KGlu buffer (120 mM K-glutamate, 20 mM K-Ac, 20 mM HEPES-KOH, 4 mM MgCl_2_ pH 7.2). Membrane sheets were prepared as described previously [32], [40] in KGlu buffer containing 10 mM EGTA. For Figs. 6, 7, 8 and S1 all steps were performed in KGlu containing 10 mM EGTA without MgCl_2_. Liposomes containing 5 mol% of ganglioside GM1 (monosialotetrahexosyl-ganglioside, Cat# 860065P, Avanti-Polar Lipids) and 95 mol% of DOPC (Cat# 850375C; 1,2-dioleoyl-sn-glycero-3-phosphocholine, Avanti-Polar Lipids) were generated by freeze-thaw cycles and extrusion. In detail, lipids were mixed, dried under Nitrogen atmosphere, resuspended in KGlu or Ringer solution achieving a final lipid concentration of 2 mg/ml and vigorously shaken for 1 h at 37°C. After 10 freeze-thaw-cycles using liquid nitrogen and a 37°C waterbath, the liposome mixture was extruded 11 times through a 400 nm filter (Cat# 800282, Whatman) and finally stored at 4°C. In all control experiments GLTP was replaced by PBS-glycerol referring to the volume of the condition with the highest applied GLTP concentration. After GLTP treatment cells or membrane sheets were washed 3 times for 5 min with Ringer or KGlu buffer, respectively. Afterwards, from the cells membrane sheets were generated.

### Staining and Microscopic Imaging Procedures

GM1 staining was performed by incubations with 1 µg/ml (membrane sheets, in KGlu) or 2 µg/ml (cells, in Ringer) recombinant CTX subunit B coupled to Alexa Fluor® 555 (Cat# C34776, Life Technologies) or Alexa Fluor® 594 (Cat# C34777, Life Technologies) for 60 min and subsequent 3 washing steps with the respective buffer for 15 min each. Finally, samples were fixed with 4% [w/v] paraformaldehyde in PBS for 45 min. For confocal analysis, to prevent CTX internalization, all steps including washing, staining and fixation were performed on ice, instead of incubation at RT for the membrane sheets. After fixation, paraformaldehyde was quenched with 50 mM NH_4_Cl in PBS for 10 min and samples were rinsed 2 times with PBS.

Whole cell samples were mounted in Mowiol embedding medium (6 g glycerol, 2.4 g Mowiol (Hoechst), 6 ml water, 12 ml 200 mM Tris, pH 7.2) containing DABCO (1,4-diazabicyclo[2.2.2]octane; Carl Roth, Germany) and imaged with a confocal Olympus FluoView1000 laser scanning microscope with an UPlanSApo × 60 NA 1.35 objective. Intact cells were first identified in the differential interference contrast (DIC) modus. Then, in a confocal section in the equatorial plane of the cells, Alexa Fluor® 555-fluorescence was excited with a 543 nm laser and emitted fluorescence was filtered between 555 and 655 nm. Image acquisition was performed with a frame size of 512 x 512 pixel applying a pixel size of 103 nm and a sampling speed of 40 µs/pixel. For analysis, images of cells in the DIC-channel were selected randomly. Linescans (three pixel width) were placed along the cell peripheries. Measurement of the mean fluorescence intensity was then performed in the Alexa Fluor® 555 channel. Background fluorescence was quantified in a rectangular region in the center of the cells and subtracted from the mean fluorescence measured at the plasma membrane.

Membrane sheets were imaged in PBS at RT with TMA-DPH (1-(4-tri-methyl-ammonium-phenyl)-6-phenyl-1,3,5-hexatriene p-toluenesulfonate, Life Technologies) to identify intact membranes (all TMA-DPH images are shown at arbitrary scaling). For imaging we used a Zeiss Axio Observer D1 fluorescence microscope. Filter sets and camera are described in [40]. Additionally, we used an Olympus IX81 microscope equipped with an MT20 illumination system in combination with the F36-500 DAPI HC-Filterset and the F36-503 TRITC HC-Filterset (AHF Analysentechnik, Tuebingen, Germany). Images were acquired with an EMCCD camera (16 µm x 16 µm pixel size, ImagEM C9100-13, Hamamatsu Photonics, Hamamatsu, Japan) and a 60 NA 1.49 Apochromat objective.

Image analysis and quantification was performed using ImageJ software. A rectangular region of interest within a membrane sheet was placed randomly in the TMA-DPH channel. The measured mean CTX fluorescence intensities were corrected for the background signals. Standard deviation/mean analysis as shown in Fig. 3 has been described previously [39].

## Supporting Information

Figure S1 **Inhibitory effect of GLTP mutations.** Experiment as in Fig. 2D, comparing the activity of wt-GLTP to the mutants GLTP-W96A and GLTP-W142A; values were normalized to wt-GLTP. Working concentrations were 1 µM (white), 5 µM (grey) and 25 µM (as in Fig. 2D; black). As shown in Fig. 1, loading is hardly increased when GLTP is raised from 5 µM to 25 µM, indicating saturation of the acceptor membrane. Accordingly, kinetic differences in transfer cycles cannot be resolved at 25 µM, whereas the strongest inhibitory effect of the mutations is observed at 1 µM. Values are given as means ± SEM (n  = 3 independent experiments; 28 - 98 membrane sheets analyzed for each experiment).
(TIF)

Figure S2 **Extraction of GM1 from HepG2 cells.** (A and B) Cells were incubated in Ringer solution with 0, 25 or 100 µM GLTP at 37°C for 30 min. Afterwards GM1 was visualized, imaged and quantified as in Fig. 1. Values are given as means ± SEM (n  = 4 independent experiments; 14 - 28 cells were analyzed for each experiment).
(TIF)
