# Supplementary figures and images for: GLTP Mediated Non-Vesicular GM1 Transport between Native Membranes
Source: PLoS One. 2013 Mar 28;8(3):e59871. doi: 10.1371/journal.pone.0059871 (PMC3610762; doi:10.1371/journal.pone.0059871)

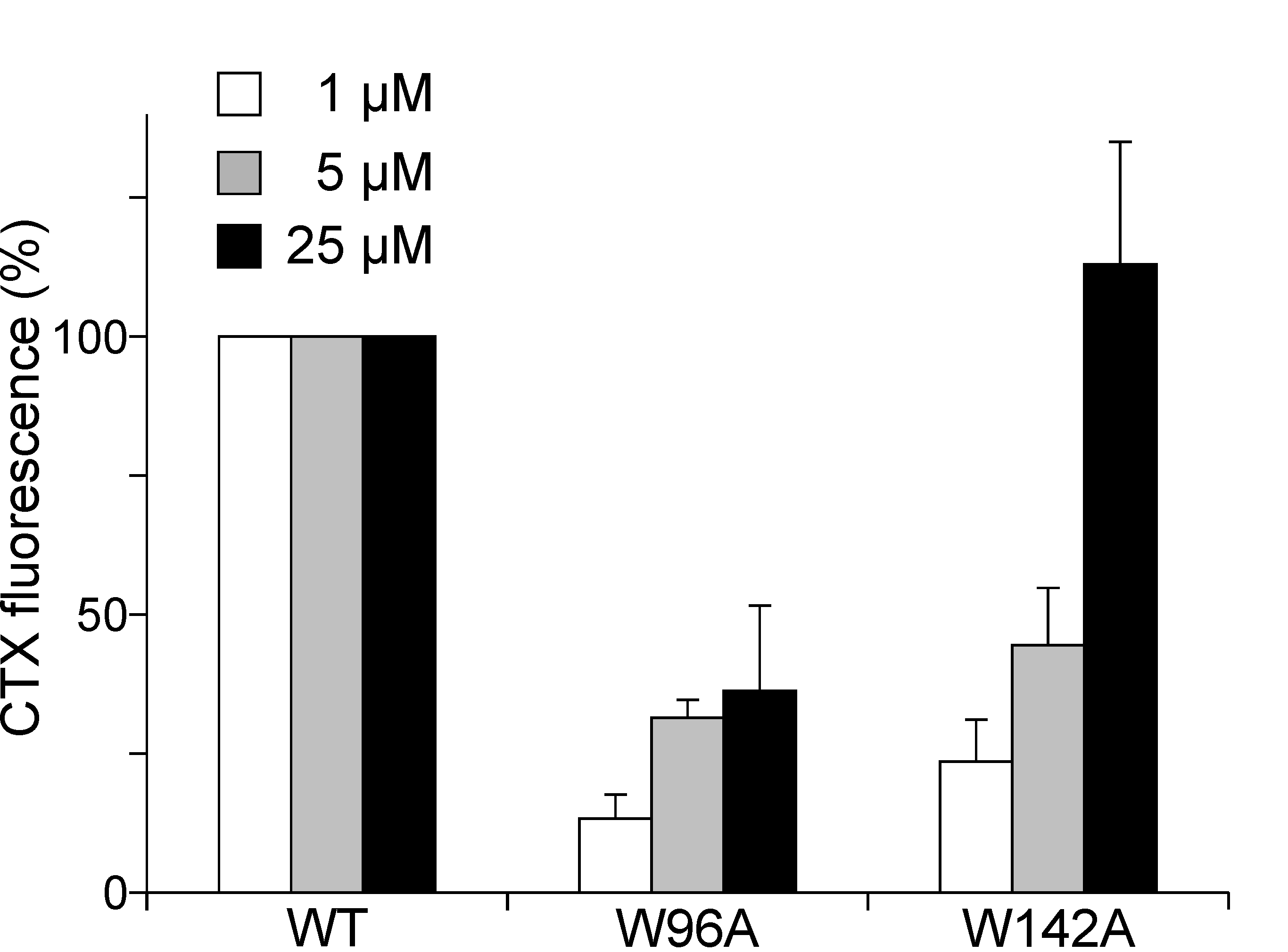

Supplement: Figure S1 — Inhibitory effect of GLTP mutations. Experiment as in Fig. 2D, comparing the activity of wt-GLTP to the mutants GLTP-W96A and GLTP-W142A; values were normalized to wt-GLTP. Working concentrations were 1 µM (white), 5 µM (grey) and 25 µM (as in Fig. 2D; black). As shown in Fig. 1, loading is hardly increased when GLTP is raised from 5 µM to 25 µM, indicating saturation of the acceptor membrane. Accordingly, kinetic differences in transfer cycles cannot be resolved at 25 µM, whereas the strongest inhibitory effect of the mutations is observed at 1 µM. Values are given as means ± SEM (n = 3 independent experiments; 28 - 98 membrane sheets analyzed for each experiment). (TIF) [file pone.0059871.s001.tif]

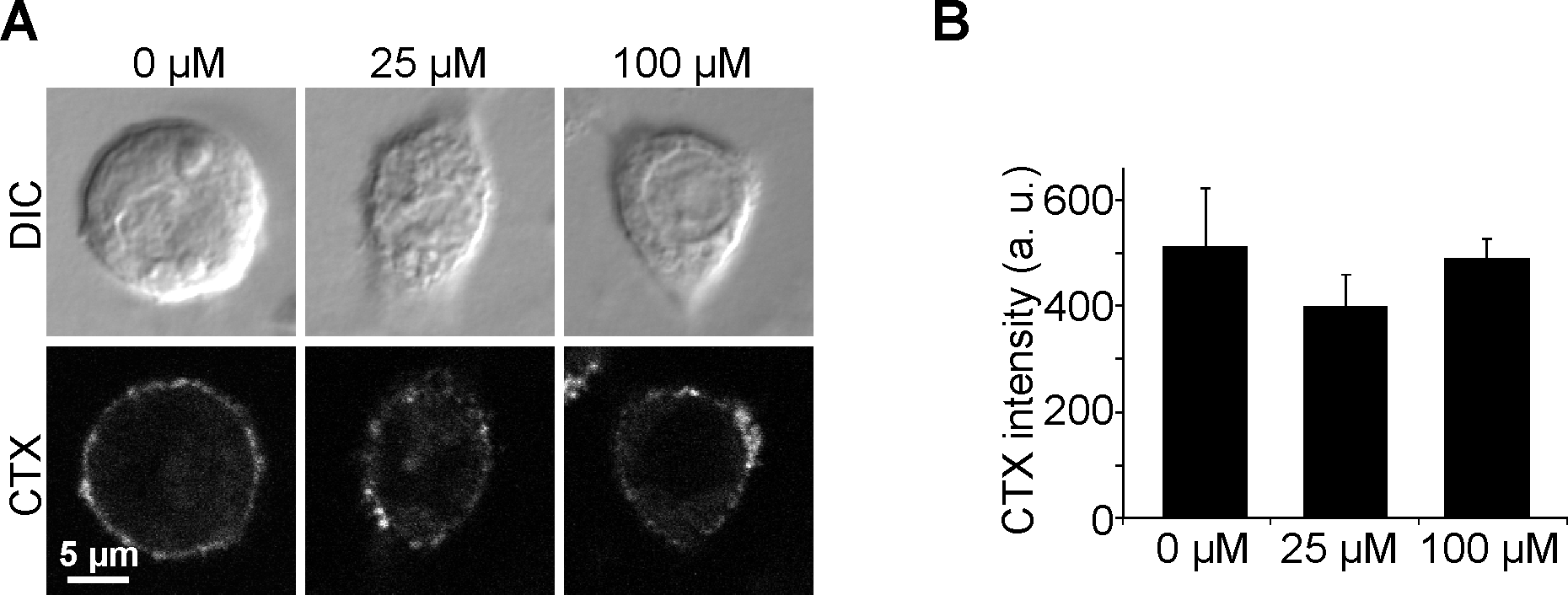

Supplement: Figure S2 — Extraction of GM1 from HepG2 cells. (A and B) Cells were incubated in Ringer solution with 0, 25 or 100 µM GLTP at 37°C for 30 min. Afterwards GM1 was visualized, imaged and quantified as in Fig. 1. Values are given as means ± SEM (n = 4 independent experiments; 14 - 28 cells were analyzed for each experiment). (TIF) [file pone.0059871.s002.tif]
